# Enhancing Immune Response and Heterosubtypic Protection Ability of Inactivated H7N9 Vaccine by Using STING Agonist as a Mucosal Adjuvant

**DOI:** 10.3389/fimmu.2019.02274

**Published:** 2019-09-27

**Authors:** Jian Luo, Xu-ping Liu, Fei-fei Xiong, Fei-xia Gao, Ying-lei Yi, Min Zhang, Ze Chen, Wen-song Tan

**Affiliations:** ^1^State Key Laboratory of Bioreactor Engineering, East China University of Science and Technology, Shanghai, China; ^2^Shanghai Institute of Biological Products, Shanghai, China

**Keywords:** H7N9, whole viron vaccine, STING, mucosal adjuvant, cross protection

## Abstract

Influenza vaccines for H7N9 subtype have shown low immunogenicity in human clinical trials. Using novel adjuvants might represent the optimal available option in vaccine development. In this study, we demonstrated that the using of the STING agonist cGAMP as a mucosal adjuvant is effective in enhancing humoral, cellular and mucosal immune responses of whole virus, inactivated H7N9 vaccine in mice. A single dose of immunization was able to completely protect mice against a high lethal doses of homologous virus challenge with an significant dose-sparing effect. We also found that intranasal co-administration of H7N9 vaccine with cGAMP could provide effective cross protection against H1N1, H3N2, and H9N2 influenza virus. Furthermore, cGAMP induced significantly higher nucleoprotein specific CD4^+^ and CD8^+^ T cells responses in immunized mice, as well as upregulated the IFN-γ and Granzyme B expression in the lung tissue of mice in the early stages post a heterosubtypic virus challenge. These results indicated that STING agonist cGAMP was expected to be an effective mucosal immune adjuvant for pre-pandemic vaccines such as H7N9 vaccines, and the cGAMP combined nasal inactivated influenza vaccine will also be a promising strategy for development of broad-spectrum influenza vaccines.

## Introduction

In March 2013, the first identified case of human infection with avian influenza A (H7N9) virus occurred in China, and as of 5 September 2018, a total of 1,567 human infections with H7N9 viruses, including at least 623 deaths, were reported during the fifth epidemic wave (1, 2). More importantly, some novel biological features of the H7N9 virus, such as the high frequency of drug-resistance, emergence of highly pathogenic outbreaks in chickens and humans were discovered in this recent fifth epidemic wave in 2017 (2–4). According to the result of the United States CDC's Influenza Risk Assessment, the avian influenza A(H7N9) virus is now ranked as the influenza virus with the highest potential pandemic risk among all influenza viruses (5).The continuous evolution of the H7N9 virus poses a long-term threat to public health, and thus it is imperative to strengthen prevention and control strategies.

Vaccination is the most effective way to prevent against seasonal and pandemic influenza caused by influenza viruses. Since the first outbreak of H7N9, different types of candidate H7N9 vaccines have been developed and are currently undergoing clinical trials. However, published clinical data demonstrated that H7N9 vaccines show poor immunogenicity in humans and using of novel adjuvants, such as MF59, AS03, immuno-stimulating complex (ISCOM), and aluminum hydroxide may have an important effect on improving vaccine immunogenicity for the uniquely low immunogenicity of this strain (6–9).

The respiratory tract mucosa is the site of infection for influenza viruses and the local immune responses on mucosal surfaces play an important role in defense against viral infection (10). Several studies revealed that the intranasal administration of inactivated vaccines, combined with an appropriate adjuvant induced well protection and cross protection against infection by both homologous and heterosubtypic viruses (11–13).

Cyclic GMP-AMP (cGAMP) is an endogenous cyclic dinucleotide catalysts synthesized by the recently discovered cyclic-GMP-AMP synthase (cGAS), which was activated by pathogen-derived cytosolic double stranded DNA. The cGAMP can be bound to the stimulator of interferon genes (STING), leading to the activation of IRF3 and induction of interferon-β, thus cGAMP functions as an endogenous second messenger in innate immune signaling by cytosolic DNA (14, 15). Both *in vivo* and *in vitro* studies suggest that cGAMP could be used as an effective adjuvant for a model antigen, like OVA and vaccines, such as porcine reproductive and respiratory syndrome virus (PRRSV) virus-like particles, and anthrax toxins (16–18). Recently, cGAMP have also been demonstrated to be an ideal adjuvant for cutaneous vaccination of influenza vaccine (19).

Besides adjuvant effect, safety issues for the cGAMP have to be considered. cGAMP is a natural metabolizable molecule in humans and is hydrolyzed quickly by ecto-nucleotide pyrophosphatase/phosphodiesterase (ENPP1) when located outside the plasma membrane, ensuring that its adjuvant activity is transient, effectively circumventing unwanted systemic inflammation (16). In addition, studies have shown that cGAMP does not cause any significant skin or acute local inflammatory responses and is not toxic to the liver or kidney (18, 19). Therefore, as a natural ligand for STING, cGAMP might be a more promising candidate adjuvant for next generation vaccines.

In terms of convenience of vaccination and the capability of inducing cross protection by mucosal immunization, a mammalian 2′, 3′-cGAMP was used as a mucosal adjuvant for inactivated whole-virion H7N9 influenza vaccine in the present study. We demonstrated that cGAMP enhances serum and mucosal antibodies, T cells, innate immune responses, as well as the protective ability of H7N9 vaccine in mice. Further, we showed that intranasal delivery of inactivated H7N9 vaccine formulated with cGAMP can induce a more robust T cell response against virus conserved epitopes that mediate cross protection against heterosubtypic influenza A viruses. Therefore, the cGAMP may be a promising vaccine adjuvant for the broad-spectrum influenza vaccines.

## Materials and Methods

### Vaccine, Viruses, Mice, and Adjuvants

An egg-derived, formalin-inactivated whole-virion H7N9 influenza vaccine based on vaccine candidate virus A/Shanghai/2/2013 H7N9 (NIBRG-267) was manufactured by Shanghai Institute of Biological Products (Figure S1). The vaccine has passed the quality control test in accordance with the requirements of Chinese Pharmacopeia (2015, Edition 3), and now is currently under phase II clinical trials. Influenza viruses used in this study included mouse adapted A/Shanghai/2/2013 (Sh2/H7N9), A/PR/8/34 (H1N1) virus, A/Guizhou/54/1989 (Gz54/H3N2), and A/Chicken/Jiangsu/7/2002 (H9N2) viruses as described in our previous studies (20, 21). Specific pathogen free (SPF) female BALB/c mice (6–8 weeks old) were purchased from Shanghai Laboratory Animal Center, China. All mice were bred in the animal resource center at Shanghai Institute of Biological Products and maintained under SPF conditions with constant temperature and humidity. The protocol for the animal study (Protocol Number: 17-1250) was approved by the laboratory animal management committee, and the laboratory animal ethics and welfare protection group of Shanghai Institute of Biological Products. All animal procedures were carried out in accordance with the animal ethics guidelines of the Chinese National Health and Medical Research Council (NHMRC). Adjuvant 2′-3′-cGAMP (Invivogen) was diluted with endotoxin-free water to a concentration of 1 mg/mL.

### Immunization and Viral Challenge

For a homologous protection study, mice were intranasally immunized once with different doses (0.015 ug, 0.15 ug, and 1.5 ug HA) of H7N9 vaccine alone or with 5 ug 2′-3′-cGAMP in a total volume of 25 ul. The 5 ug 2′-3′-cGAMP immunized and an unimmunized group was used as an adjuvant control group and negative control group respectively. Three weeks after immunization, mice were anesthetized and challenged intranasally with 20 μl of the viral suspension containing 40 × LD_50_ of A/Shanghai/2/2013 (Sh2/H7N9) virus. For a heterosubtypic protection study, mice were intranasally immunized with either 1.5 ug HA of H7N9 vaccine alone or with 5 ug 2′-3′-cGAMP twice on day 0 and 21. Three or six weeks (for long term protection) after the last immunization mice were anesthetized and challenged intranasally with 20 μl of the viral suspension containing 5 × LD_50_ of A/PR/8/34 (H1N1) or A/Guizhou/54/1989 (Gz54/H3N2) or A/Chicken/Jiangsu/7/2002 (H9N2) influenza virus. Survival and body weight loss were monitored for 2 weeks post virus challenge.

### Specimens Preparation

Five mice from each group were randomly chosen for sample collection at a predetermined time after immunization or virus challenge (see Results). The sera were collected from the blood and used for serum IgG, Hemagglutination inhibition (HI) antibodies assays. The spleens were taken out by sterile forceps to prepare PBMC. Mouse lungs were collected and homogenized in 1.5 mL of PBS containing Penicillin-Streptomycin (Gibco, USA) by an electric homogenizer Tissuelyser-24 (Jingxin, Shanghai, China). Finally, a syringe needle with 1 mL of PBS was inserted three times into the nasopharynx to collect the nasal wash. The lung homogenates and nasal wash were centrifuged to remove cellular debris.

### Antibody Assays

The titers of virus specific IgG and IgA of mice 3 weeks after a single dose immunization were measured by enzyme-linked immunosorbent assay (ELISA), which was performed using a series of reagents consisting of: firstly, 5 μg/mL of inactivated whole-virion H7N9 vaccine for plate coating; secondly, serial 2-fold dilutions of sera or nasal wash or lung homogenates; thirdly, goat anti-mouse IgG Ab (γ-chain specific) (KPL) or goat anti-mouse IgA (α-chain specific) (KPL) conjugated with horseradish peroxidase (HRP); and finally, the substrate 3, 3′, 5, 5′-Tetramethylbenzidine (TMB). The amount of chromogen produced was measured based on absorbance at 450 nm. Ab-positive cut-off values were set as means+2×SD of PBS control group. An ELISA Ab titer was expressed as the highest serum dilution giving a positive reaction.

The hemagglutination inhibition (HAI) antibody titers of sera against different virus strains by a one or two doses immunization was determined by HI assay. Briefly, sera were pretreated with a receptor destroying enzyme (Diho, China) for 20 h at 37°C and then inactivated at 56°C for 30 min; 2-fold serial dilutions of 50 μl pretreated sera and positive control sera were incubated with an equal volume of 4 HA units of selected virus antigen for 1 h at room temperature and then 50 μl of a 1% suspension of chicken red blood cells (RBC) were added. After 30 min of incubation at room temperature, the HI titers were determine by the highest dilution of sera that completely inhibits the agglutination of the chicken RBC. The limit of detection for this assay is a 1:10 dilution.

### ELISpot Assays

Virus or nucleoprotein (NP) specific IFN-γ secreting splenocytes of immunized mice was determined by ELISpot assay as described in our previous study (20). For detecting virus specific IFN-γ secreting splenocytes, 10 μg/mL of H7N9 influenza vaccine was used as a stimulant. As well, an H-2d-restricted NP class I peptide and a pool of three H-2d restricted class II peptides as described in our previous study were used as stimulatory agents for detection of NP specific IFN-γ secreting CD8^+^ T cells and CD4^+^ T cells, respectively. The number of virus or peptide-reactive cells was represented as spot forming cells per 10^6^ splenocytes and was calculated by subtracting spot numbers in control peptide (HIV pol peptide ILKEPVHGV) wells from that in NP specific peptide (or H7N9 influenza vaccine) containing wells. The number of peptide-reactive cells was represented as spot forming cells per 10^6^ splenocytes.

### Analyses of Lung Cytokines and Cytotoxic Effector Molecules

Lung homogenates from five mice in each group were collected at 24 h post a single dose immunization for detection of cytokines (IL-6, TNF-α, IL-1β) by mouse cytokine MILLIPLEX® MAP kits (MCYTOMAG-70K, Millipore) according to the manufacturer's protocol. Measurements were performed using the Bio-Plex MAGPIX Multiplex reader. Concentrations of IFN-γ and Granzyme B in whole lung homogenates of mice post a heterosubtypic influenza A viruses challenge were determined by quantikine mouse IFN-γ ELISA Kit (88-8314-22, invitrogen) and Mouse Granzyme B ELISA Kit (GWB-SKR178,GENWAY) according to the manufacturer's protocol.

### Virus Titrations

Virus titration was performed as described previously (21). Lung homogenates were serially diluted 10-fold and loaded on confluent MDCK cells, which were subsequently incubated in the growth medium and tested for hemagglutination 72 h later. The virus titer of each specimen, expressed as the 50% tissue culture infection dose (TCID_50_), was calculated by the Reed-Muench method.

### Statistics

GraphPad Prism 5 software was used to perform statistical analyses. The survival rates of the mice in the test and control groups were evaluated by Log-rank (Mantel-Cox) test; the results of serum and mucosal antibody titers, lung virus titers and cytokine response were evaluated by one-way ANOVA and Tukey's multiple comparison test; if the *p*-value was < 0.05, the difference was considered significant.

## Results

### cGAMP Adjuvanted Vaccine Offers Improved Protection Against a High Lethal Dose Challenge of Homologous H7N9 Virus

One twenty mice were randomized into 8 groups (A-H), with 15 mice in each group. Mice were all immunized intranasally (i.n.) once with various doses of whole-virion H7N9 influenza vaccine alone or in combination with cGAMP as an adjuvant, respectively; The control group was not immunized. All mice were then i.n. challenged with a high lethal dose (40 × LD_50_) of mouse adapted A/Shanghai/2/2013 (Sh2/H7N9) viral suspension 3 weeks post-immunization. The lung hemogenate of five mice in each group was prepared and used for virus titration on day 3 after challenge. The survival rates and the body weight losses of the remaining 10 mice in each group were monitored for 2 weeks after the challenge.

**Figure 1:**
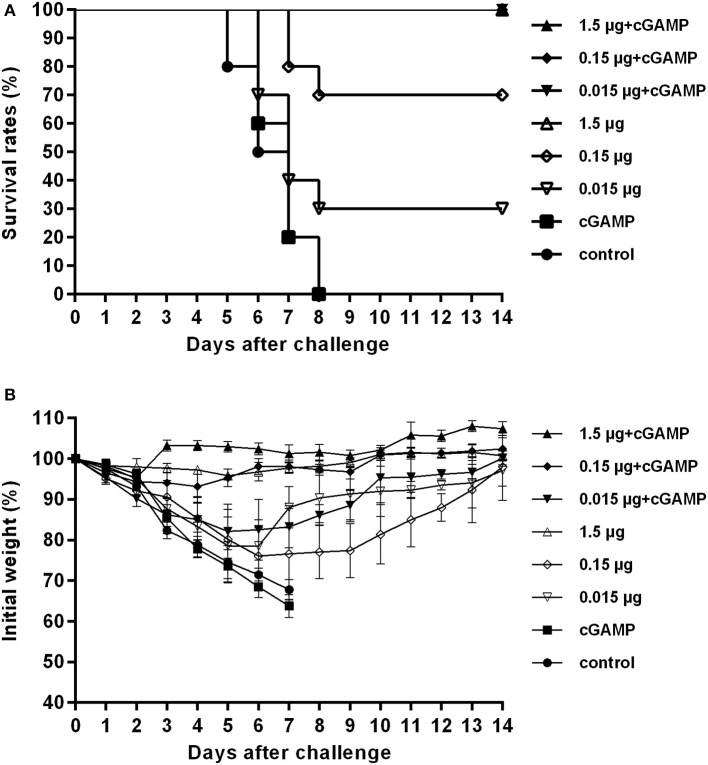
cGAMP adjuvanted vaccine offers improved protection against a high lethal dose challenge of homologous virus. Mice were intranasally immunized once with various doses of whole-virion H7N9 influenza vaccine with or without cGAMP. Three weeks after immunization, mice were challenged intranasally with a high lethal dose (40 × LD_50_) of mouse adapted Sh2/H7N9 influenza virus. Survival rates **(A)** and body weight changes **(B)** of mice were measured daily for 2 weeks after challenge.

The results presented in Table 1 showed that the mice in the control group and the group immunized with cGAMP alone suffered a rapid reduction in body weight post virus challenge, and failed to provide any protection. The protection efficiency offered by H7N9 influenza vaccine alone was dependent on vaccine dosage, only the high dosage group could provide full protection against a high lethal dose challenge of influenza H7N9 virus, while the low and medium dosage groups provided 30 and 70% protection, respectively. In contrast, all the adjuvanted vaccine immunized groups could provide effective protection, regardless of the dosage (Figure 1A). Moreover, these mice showed relatively mild weight loss and faster recovery times following the challenge, as compared to the groups without cGAMP (Figure 1B).

**Table 1:** Protection against a high lethal dose challenge of homologous influenza virus in mice by intranasal administration of whole-virion H7N9 influenza vaccine with or without cGAMP.

**Group**	**Dose and adjuvant**	**Lung virus titer^a^ (log_10_TCID_50_/ml)**	**Survival rate (No. of survivors/no. tested)**
A	1.5μg + cGAMP	Undetected^b^^,^ ^c^	10/10^b^
B	1.5μg	Undetected^b^	10/10^b^
C	0.15μg + cGAMP	5.15 ± 0.42^b^^,^ ^c^	10/10^b^
D	0.15μg	7.60 ± 0.82^b^	7/10^b^
E	0.015μg + cGAMP	8.10 ± 0.93^b^^,^ ^c^	10/10^b^^,^ ^c^
F	0.015μg	11.60 ± 0.55	3/10
G	cGAMP	12.70 ± 0.62	0/10
H	control	12.45 ± 0.65	0/10

*Mice were intranasally immunized once with various doses of whole-virion H7N9 influenza vaccine with or without cGAMP. Three weeks after the last immunization, mice were challenged with a high lethal dose (40 × LD_50_) of mouse adapted A/Shanghai/2/2013 (Sh2/H7N9) virus. Lung hemogenate from 5 mice in each group were collected 3 days post-infection for titration of lung virus. The survival rates of mice 2 weeks post-infection were determined*.

a *Results are expressed as mean ± SD of five tested mice in each group*.

b *Displays significant difference compared with mice in control groups (P < 0.05)*.

c *Displays significant difference compared with mice in the corresponding non- adjuvanted groups (P < 0.05)*.

The results of lung virus titers were also shown in Table 1. The lung virus titer of immunized mice had a dosage dependent decreasing trend. Furthermore, the virus titer in cGAMP adjuvanted group was significantly lower than that of the non-adjuvanted group within the same dosage (*P* < 0.05). The obtained results indicate that a single dose immunization with inactivated whole-virion H7N9 vaccine plus cGAMP provided increased protection over vaccine alone, and reduced the viral load in the lungs after a high lethal dose challenge of a homologous H7N9 influenza virus.

### cGAMP Adjuvant Enhances the Mucosal and Systemic Antibody and T Cell Responses of Whole-Virion H7N9 Influenza Vaccine

Forty mice were randomized into 8 groups, with 5 mice in each group, mice were immunized as described above. The titers of virus specific IgG and hemagglutination inhibition(HAI) antibodies in serum, virus specific IgA in nasal wash and lung hemogenate were detected at week 3 after immunization. The ELISpot was conducted to detect the cellular immune based on the amount of IFN-γ secreting splenocytes of immunized mice after being stimulated with whole-virion H7N9 influenza vaccine *in vitro*.

As shown in Table 2, all groups except the cGAMP group and the control group had an obvious serum antibody response in a dose-dependent manner, among which the serum antibody responses induced by the vaccine plus cGAMP were significantly higher than those in the vaccine alone group with the same dosage (*P* < 0.05). We also evaluated the serum HAI antibody titers, which have been correlated with the protective efficacy of influenza vaccines. The HAI antibody titers against the H7N9 virus elicited by the cGAMP adjuvanted vaccine were remarkably higher than those elicited by the vaccine alone. These results indicated that cGAMP was able to enhance the virus-specific antibody as well as the HAI antibody responses in mouse serum induced by whole-virion H7N9 influenza vaccine.

**Table 2:** Serum and mucosal antibody responses in mice by intranasal administration of inactivated H7N9 vaccine with or without cGAMP adjuvant.

**Group**	**Dose and adjuvant**	**Virus-specific antibody or HAI antibody titer**			
		**Virus-specific antibody titer (ELISA, 2^n^)^a^**			**HAI antibody titer^a^**
		**Serum IgG**	**Nasal wash IgA**	**lung hemogenate IgA**	**Serum HAI**
A	1.5μg + cGAMP	8.60 ± 0.56^b^^,^ ^c^	5.40 ± 0.55^b^^,^ ^c^	3.60 ± 0.55^b^^,^ ^c^	448 ± 175.27^b^^,^ ^c^
B	1.5μg	7.00 ± 0.71^b^	2.60 ± 1.14^b^	2.20 ± 0.45^b^	64 ± 21.91^b^
C	0.15μg + cGAMP	6.60 ± 1.14^b^^,^ ^c^	4.00 ± 1.00^b^^,^ ^c^	2.20 ± 0.84^b^^,^ ^c^	48 ± 17.89^b^^,^ ^c^
D	0.15μg	4.80 ± 0.84^b^	1.80 ± 0.84^b^	ND	20 ± 0.00^b^
E	0.015μg +cGAMP	4.20 ± 0.84^b^^,^ ^c^	ND	ND	16 ± 5.48^b^^,^ ^c^
F	0.015μg	2.80 ± 0.84^b^	ND	ND	ND
G	cGAMP	NT	NT	NT	NT
H	control	NT	NT	NT	NT

*Mice were intranasally immunized once with various doses of whole-virion H7N9 influenza vaccine with or without cGAMP. Three weeks after the immunization, serum, nasal wash, and lung hemogenate specimens of 5 mice in each group were prepared. The titers of virus specific IgG and hemagglutination inhibition (HAI) antibodies in serum, virus specific IgA in nasal wash and lung hemogenate were detected by ELISA or HI assay. An ELISA Ab titer was expressed as the highest dilution giving a positive reaction. ND, not detected; NT, not tested*.

a *Results are expressed as mean ± SD of five tested mice in each group*.

b *Displays significant difference compared with mouse in control groups (P < 0.05)*.

c *Displays significant difference compared with mouse in the corresponding non-adjuvanted groups (P < 0.05)*.

Since the influenza virus enters the body through the respiratory tract, determining the presence of antibodies in secretory mucosal samples is important. As also shown in the Table 2, although the IgA antibody in the nasal wash and lung hemogenate was undetected in the low dose of 0.015 μg in both adjuvant and non-adjuvanted groups, the medium and high dosage of the vaccine supplemented with cGAMP could induce high levels of mucosal IgA, which were significantly higher than those in the same dosage of non-adjuvanted groups (*P* < 0.05), indicating cGAMP can play a role as an effective adjuvant for inducing enhanced mucosal virus specific antibody responses against influenza virus.

**Figure 2:**
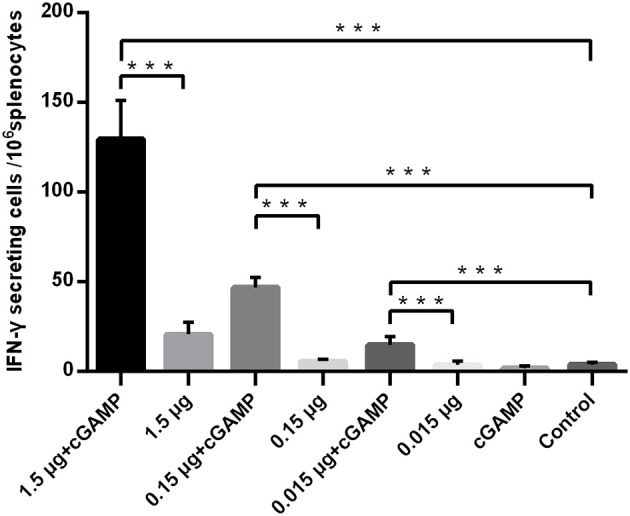
cGAMP adjuvant enhances the T cell responses of whole-virion H7N9 influenza vaccine. Mice were intranasally immunized once with various doses of whole-virion H7N9 influenza vaccine with or without cGAMP. The amount of IFN-γ secreting splenocytes of immunized mice after stimulated with whole-virion H7N9 influenza vaccine was measured by ELISpot three weeks after immunization. Data shown as mean numbers of spot-forming cells (SFCs) ± SD. *n* = 5 per immunized group. Each sample was tested in triplicates. ^***^P < 0.001 (one-way ANOVA and Tukey's multiple comparison test).

The T cell response was analyzed by an IFN-γ ELISpot assay, and the results are shown in Figure 2. A trend toward increased IFN-γ secreting was noted in mice i.n. immunized with non-adjuvanted H7N9 compared with the control, however, this trend was not statistically significant (P>0.05), suggesting that i.n. administration of a single dose of inactivated H7N9 vaccine did not induce a significant cellular immune response, as least at the dosage used in this study. By contrast, the mice immunized with the H7N9 vaccine plus cGAMP developed a significant number of IFN-γ secreting splenocytes, with an average of seven times more than the vaccine alone groups. In conclusion, intranasal administrated of whole-virion H7N9 influenza vaccine with cGAMP as a mucosal adjuvant in mice could induce an increased systemic and mucosal antibody and T cell responses that confer better protection against a high lethal dose challenge of homologous influenza virus.

### Intranasal Administration of cGAMP Increases Expression of Innate Immunity Cytokines of Mice in Lungs

**Figure 3:**
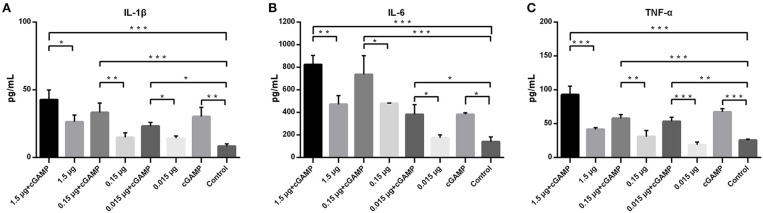
cGAMP adjuvant enhances the expression of mouse lung innate immune cytokines. Mice were intranasally immunized once with various doses of whole-virion H7N9 influenza vaccine with or without cGAMP. The lung homogenates of immunized mice were collected for IL-1β, IL-6, and TNF-α measurement by ELISA 24 h after immunization. Data shown as mean ± SD. *n* = 5 per immunized group. Each sample was tested in triplicates. ^***^P < 0.001, ^**^P < 0.01 and ^*^P < 0.05 (one-way ANOVA and Tukey's multiple comparison test).

To assess the ability of cGAMP in influencing expression of innate immunity cytokines, forty mice were randomized into 8 groups, with 5 mice in each group, mice were immunized as described above. Lung homogenates of mice in each group were collected at 24 h post-immunization for detection of the expression of proinflammatory cytokines, including IL-1β, IL-6, and TNF-α. As shown in the Figure 3, intranasal immunization of inactivated H7N9 vaccine in a relatively high-dose alone could induce a certain level expression of IL-1β, IL-6, or TNF-α in mice lung homogenates (compared to the control group), while all the cGAMP adjuvanted groups induced a higher expression of innate immunity cytokines, as compared to the groups without cGAMP after 24 h (*P* < 0.05). The greater expression of IL-1β, IL-6 or TNF-α was directly associated to the presence of cGAMP. However, the expression of IL-1β, IL-6, or TNF-α did not cause obvious pathological and histological change in lung tissues of mice (Figure S2). This result suggests that the enhancement of mucosal and systemic immune responses to whole-virion H7N9 influenza vaccine by cGAMP was very likely due to the innate immune recognition of cGAMP and the expression of innate immune mediators.

### cGAMP Adjuvanted Vaccine Induces Improved Cross Protection Against a Lethal Dose Challenge of the Heterosubtypic Virus

Intranasal administration of a whole inactivated influenza virus vaccine was proved to be a promising way to induce a broad spectrum of heterosubtypic immunity against influenza A virus. To explore whether the addition of cGAMP could enhance the cross protection ability of the whole inactivated H7N9 influenza vaccine against a heterosubtypic influenza virus infection, a two-dose immunization regimen was used since a single dose of 1.5 μg vaccine plus cGAMP could not provide good protection against a heterosubtypic virus challenge (data not shown). Mice were intranasally immunized with either 1.5 ug HA of H7N9 vaccine alone or with 5 ug 2′-3′-cGAMP twice on day 0 and 21, and an unimmunized group was used as an negative control group. Three weeks after the last immunization, all the mice were i.n. challenged with 5 × LD_50_ of mouse adapted A/Puerto Rico/8/34 (H1N1), A/Guizhou/54/1989(Gz54/H3N2) and A/ Chichen/Jiangsu/11/ 2002 (H9N2) viral suspension (as shown in Table 3). Compared to the control group, a full protection against a lethal challenge of all the three virus strains was obtained in the adjuvanted vaccine immunized group, while the protection rates against H1N1, H3N2, and H9N2 were 60%, 50 and 60% respectively in the non-adjuvanted vaccine groups. Moreover, the lung virus titers were considerably reduced in mice immunized with the whole inactivated vaccine plus cGAMP, in comparison to mice that were vaccinated with the vaccine alone on day 3 post challenged with any of the heterosubtypic viruses. These collective results show that i.n administration of whole inactivated H7N9 vaccine in combination with cGAMP can significantly reduce the lung virus load, and confer broad spectrum protection against heterosubtypic influenza A viruses.

**Table 3:** Protection against a lethal dose challenge of the heterosubtypic viruses in mice by intranasal administration of whole-virion H7N9 influenza vaccine combined with cGAMP.

**Group**	**Dose and adjuvant**	**A/PuertoRico/8/1934 (H1N1)**		**A/Guizhou/54/1989(Gz54/H3N2)**		**A/Chicken/JiangSu/07/2002 (H9N2)**	
		**Lung virus titer^a^ (log_10_TCID_50_/ml)**	**No. of survivors/no. tested**	**Lung virus titer^a^ (log_10_TCID_50_/ml)**	**No. of survivors/no. tested**	**Lung virus titer^a^ (log_10_TCID_50_/ml)**	**No. of survivors/no. tested**
A	1.5μg + cGAMP	5.44 ± 0.30^b^^,^ ^c^	10/10^b^^,^ ^c^	6.14 ± 0.62^b^^,^ ^c^	10/10^b^^,^ ^c^	6.25± 0.38^b^^,^ ^c^	10/10^b^^,^ ^c^
B	1.5μg	9.19 ± 0.31^b^	6/10^b^	9.00 ± 0.46^b^	5/10^b^	7.00± 0.29^b^	6/10^b^
C	Control	10.13 ± 0.52	0/10	11.25 ± 0.35	1/10	8.05± 0.37	0/10

*Mice were intranasally immunized with two doses of 1.5 ug HA of whole-virion H7N9 influenza vaccine with or without 5 ug 2′-3′-cGAMP on day 0 and 21. Three weeks after the last immunization, mice were challenged with a lethal dose (5 × LD_50_) of mouse adapted A/Puerto Rico/8/34 (H1N1), A/Guizhou/54/1989(Gz54/H3N2) and A/ Chichen/Jiangsu/11/ 2002 (H9N2) influenza virus. Lung hemogenate from 5 mice in each group were collected 3 days post-infection for titration of lung virus. The survival rates of mice 2 weeks post-infection were determined*.

a *Results are expressed as mean ± SD of five tested mice in each group*.

b *Displays significant difference compared with mice in control groups (P < 0.05)*.

c *Displays significant difference compared with mice in the corresponding non-adjuvanted groups (P < 0.05)*.

### cGAMP Enhanced Cross-Reactive T Cell Response Against Virus Conserved Protein May Be Correlated With the Cross Protection

**Figure 4:**
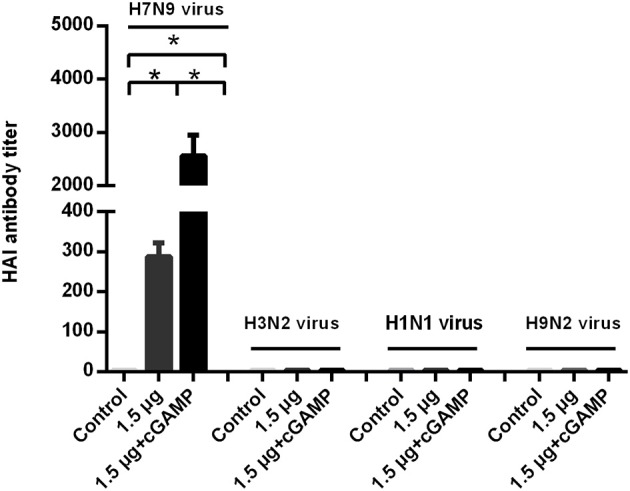
cGAMP adjuvanted H7N9 influenza vaccine does not induce detectable cross-reactive hemagglutination inhibition antibodies against heterosubtypic virus. Mice were intranasally immunized with two doses of 1.5 ug HA of whole-virion H7N9 influenza vaccine with or without cGAMP on day 0 and 21. Three weeks after immunization, serum HI antibodies against homologous and heterosubtypic of H3N2, H1N1, H9N2 viruse were determined by HI assay. Data shown as mean titer ± SD. *n* = 5 per immunized group. Each sample was tested in triplicates. ^*^P < 0.05 (one-way ANOVA and Tukey's multiple comparison test).

We examined whether it was possible to induce broad-spectrum cross-reactive HAI antibodies by intranasal administration of inactivated H7N9 in combination with cGAMP adjuvant. Mice were intranasally immunized as described above. The serum HAI antibody titers against the homo- and heterologous viruses were detected HI assay in 3 weeks after the last immunization. The results showed that a two-dose regimen of the H7N9 vaccine immunization could induce a higher level of HAI antibodies against homologous viruses, which was significantly higher than that induced by a single immunization with the same dose. In addition, the immuno-enhancing effect of cGAMP was also observed in the cGAMP adjuvanted vaccine group. However, neither the adjuvant nor the non-adjuvant group could induce detectable cross-reactive HAI antibodies against the H1N1, H3N2 and H9N2 virus (Figure 4). A further serum transfer study showed that transferring immune serum from cGAMP adjuvant vaccine immunized mice alone did not confer cross protection against a heterosubtypic virus to recipient mice (data not shown).

**Figure 5:**
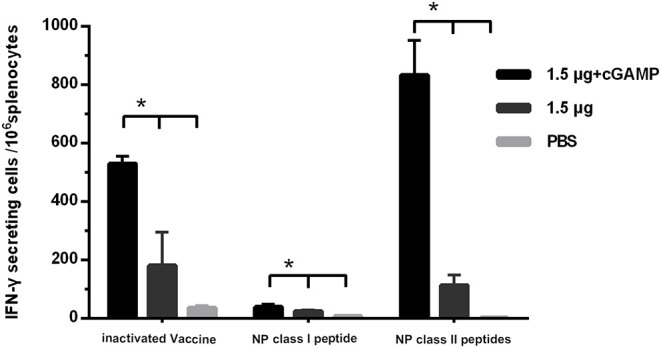
cGAMP adjuvant enhances the T cell response against virus conserved protein. Mice were intranasally immunized with two doses of 1.5 ug HA of whole-virion H7N9 influenza vaccine with or without cGAMP on day 0 and 21. The amount of IFN-γ secreting splenocytes of immunized mice after stimulated with whole-virion H7N9 influenza vaccine, NP derived MHC-I epitope peptide or a pool of three NP derived MHC-II epitope peptides was measured by ELISpot three weeks after immunization. The splenocytes of immunized mice were isolated for IFN-γ measurement by ELISpot. Data shown as mean numbers of spot-forming cells (SFCs) ± SD. *n* = 5 per immunized group. Each sample was tested in triplicates. ^*^P < 0.05 (one-way ANOVA and Tukey's multiple comparison test).

Since we did not detect cross-reactive HAI antibodies that could mediate cross protection in the immunized mice, the T cell responses against the conserved internal antigens nucleoprotein (NP) of the virus which has been proven to be able to mediate cross protection against heterosubtypic influenza virus was detected by IFN-γ ELISpot assay. The splenocytes of mice were isolated 3 weeks after the last immunization, and were, respectively, stimulated with whole inactivated influenza virus vaccine, a NP derived MHC-I epitope peptide and a pool of three NP derived MHC-II epitope peptides, which were corresponded to detection of virus specific T cell response, NP specific IFN-γ secreting CD8^+^ T cell response and NP specific IFN-γ secreting CD4^+^ T cell response. The results are shown in Figure 5. The considerable amounts of virus specific IFN-γ secreting T cells, NP specific IFN-γ secreting CD4^+^ and CD8^+^ T cells were induced by two doses of i.n immunization with the whole inactivated H7N9 vaccine. As expected, the cGAMP could effectively increase the number of NP specific IFN-γ secreting CD4^+^ and CD8^+^ T cells induced by the whole inactivated H7N9 vaccine.

**Figure 6:**
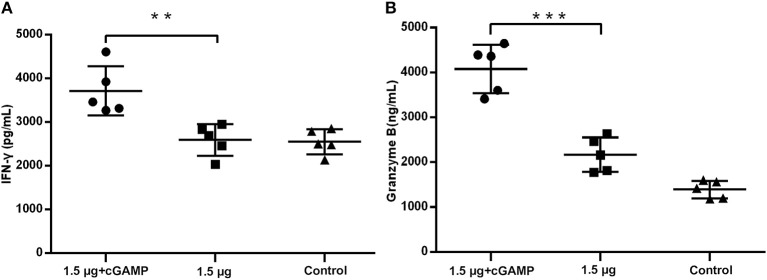
cGAMP adjuvant upregulates production of cytotoxic T cell related cytokines in lung of mice post virus challenge. Mice were intranasally immunized with two doses of 1.5 ug HA of whole-virion H7N9 influenza vaccine with or without cGAMP on day 0 and 21. Three weeks after the last immunization, mice were challenged intranasally with a lethal dose (5 × LD50) of heterosubtypic A/PR/8/34 (H1N1) influenza virus. The concentration of IFN-γ **(A)** and granzyme B **(B)** in the whole lung homogenates of mice at three days post virus challenge were measured by ELISA. Data shown as mean ± SD. n = 5 per immunized group. Each sample was tested in triplicates. ^***^P < 0.001, ^**^P < 0.01 (one-way ANOVA and Tukey's multiple comparison test).

We further elevated the expression of IFN-γ and granzyme B, which were used as the markers of activated cytotoxic T cells, in the lung homogenates of mice at 3 day post a lethal dose challenge with the heterosubtypic virus [A/PR/8/34 (H1N1)]. The results in Figure 6 showed that the mice i.n. immunized with cGAMP adjuvanted vaccine had significantly higher levels of IFN-γ and granzyme B expression in response to the viral challenge than those in the control and the non-adjuvant group. This indicated that there were more activated cytotoxic T cells recruited to the infection sites (lung tissue and nasal mucosa), mediating viral clearance in early stage post infection. Based on the above results, we speculate that the enhanced cross-reactive NP, or other virus conserved protein specific CD4^+^ and CD8^+^ T cell response induced by i.n administration of inactivated H7N9 vaccine with GAMP adjuvant may be closely related to cross protection in the absence of cross-neutralizing antibodies.

### cGAMP Adjuvanted Influenza Vaccine Provides Long-Term Cross Protection Against Heterosubtypic Influenza a Viruses Challenge

**Figure 7:**
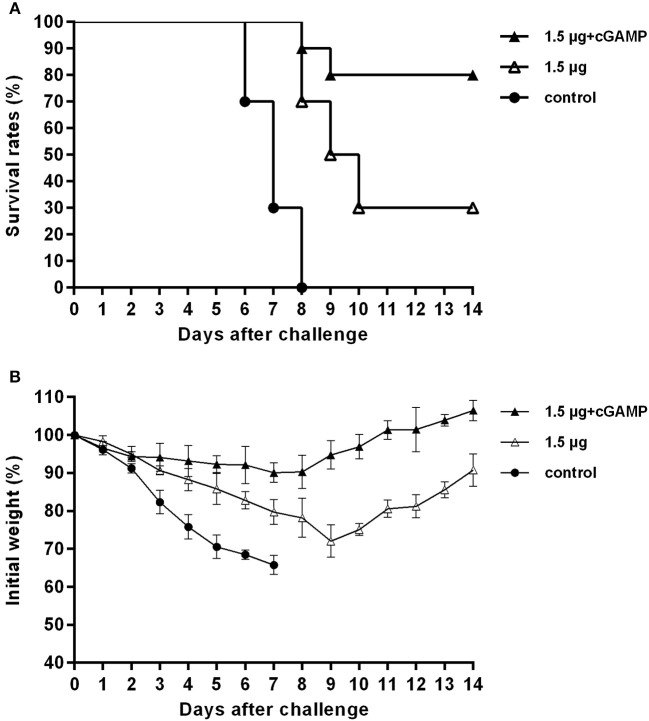
cGAMP adjuvanted influenza vaccine provides long-term cross protection against heterosubtypic influenza A viruses challenge. Mice were intranasally immunized with two doses of 1.5 ug HA of whole-virion H7N9 influenza vaccine with or without cGAMP on day 0 and 21. Six months after the last immunization, mice were challenged intranasally with a lethal dose (5 × *LD*_50_) of heterosubtypic A/PR/8/34 (H1N1) influenza virus. Survival rates **(A)** and body weight changes **(B)** of mice after being challenged with a lethal dose of heterosubtypic influenza virus.

We investigated the long-term cross protection ability against heterosubtypic influenza A virus challenge after i.n. immunized of mice with inactivated H7N9 vaccine by using cGAMP as an adjuvant. Thirty mice were randomized into 3 groups, mice were immunized with two doses of 1.5 μg inactivated whole-virion H7N9 influenza vaccine, either with or without 5 ug 2′-3′-cGAMP as an adjuvant, and an unimmunized group was used as an negative control group. Six months after the last immunization, the mice were challenged with 5 × *LD*_50_ of A/Puerto Rico/8/34 (H1N1) influenza virus, and survival rates were observed for 2 weeks. The results presented in Figure 7 indicated that i.n. immunized mice with inactivated H7N9 vaccine plus cGAMP provided up to 80% protection against a heterosubtypic influenza vchallenge in mice, which was significantly higher than the non-adjuvant group with a protection rate of 30% (*P* < 0.05), while the control group failed to provide any protection and the body weight of mice continued to decline, resulting in all mice being dead within 8 days after the challenge. Moreover, the adjuvant group showed relatively mild weight loss and faster recovery following challenge compared to the groups without cGAMP. These results strongly suggest that immunization with a cGAMP adjuvanted influenza vaccine can provide more effective and longer-term protection against lethal challenges of heterosubtypic influenza virus.

## Discussion

Currently, the epidemiological and risk assessments indicate a strong potential for the H7N9 virus to pose a public health risk (5). The development of effective vaccines is of great importance for the prevention and control of a possible H7N9 pandemic. Current influenza vaccines are usually non-adjuvanted, but the addition of an adjuvant may improve vaccine immunogenicity and permit dose-sparing, which may be critical for managing vaccine supplies during influenza pandemics (22). Previous studies have confirmed that the immunogenicity of H7N9 virus is relatively low in humans, and the usage of many different types of novel adjuvants may be necessary to improve the immunogenicity of H7N9 vaccines (9, 23, 24).

In this study, we used the novel STING agonist cGAMP as an adjuvant for the whole virus inactivated H7N9 vaccine. The results showed that cGAMP can effectively enhance the vaccine-induced serum-specific antibody and HAI antibody response in intranasally immunized mice. The antibody response induced by utilizing a cGAMP adjuvant achieved a dose-sparing effect of about 10 times higher than that of vaccine alone. That is, the serum antibody levels induced by 0.015 μg with cGAMP and 0.15 μg with cGAMP groups were comparable to the serum antibody levels induced by the 0.15 μg and 1.5 μg none adjuvanted vaccine groups, respectively. This finding may be of great value for the application of pre-pandemic influenza vaccines such as H7N9 vaccines (22). More importantly, we demonstrated that the level of mucosal antibodies induced by the vaccine supplemented with cGAMP adjuvant was significantly higher than that of the unadjuvanted group, which may play a decisive role in the first-time antiviral protection. In addition, the cGAMP was also shown to be effective in enhancing vaccine induced T cell immune responses, represented by higher induction of a strongly enhanced IFN-γ response in splenocytes from immunized mice.

The cGAMP acts as an agonist that activates the STING signaling pathway and the downstream NF-κB and IRF3 pathways to induce cytokine production to further promote adaptive immune responses via different molecular mechanisms (15, 18, 25). By examining the levels of innate immune-related cytokines in the lungs of mice at an early time post immunization, we found that intranasal administration of cGAMP can induce and increase the production of innate immune-related cytokines in lung tissues, which is consistent with previous reports by other groups using intramuscular, sublingual and skin immunization routes (16, 18). Given the fact that cGAMP acts as a natural small molecule, with its structure of phosphoric acid containing diester bonds that are easily degraded by phosphodiesterase *in vivo* (19, 26), the role of cGAMP is functional for only a short time without side effects. It is theorized that perhaps the moderate expression of these innate immune-related cytokines is important for vaccine-induced immune enhancement. Our results indicate that it is the enhanced humoral, cellular and mucosal immune responses induced by the cGAMP vaccine that protect mice during high lethal viral challenges.

The use of traditional inactivated influenza vaccines to induce broad-spectrum immune responses with novel immunization adjuvants or immunization strategies is one of the directions for development of broad-spectrum influenza vaccines. Several studies have demonstrated that intranasal immunization of inactivated influenza vaccine induces both humoral and cell-mediated immunity, suggesting that either or both of them might contribute to cross protection (11, 13, 27, 28). However, previous studies using mice intranasally vaccinated with inactivated influenza vaccines suggested that heterosubtypic immunity can occur in the absence of cross-neutralizing antibodies (29). In the present study, we also found that mice immunized intranasally with two doses of cGAMP adjuvanted H7N9 influenza vaccine did not induce cross-reactive hemagglutination inhibition antibodies against H1N1, H3N2 and H9N2 influenza viruses, but could effectively protect mice against lethal challenge of these heterosubtypic influenza viruses.

Since our previous studies have shown that mice intranasally immunized with a recombinant internal conserved NP protein in combination with mucosal immune adjuvant (Cholera toxin B or C48/80) could produce better cross protection against a heterogeneous influenza virus challenge, and of which, the cellular immune response targeting these internal conservative antigens were regarded as the main factor mediating this cross protection against influenza (30, 31). In order to investigate the potential immune mechanism that mediate cross protection against heterosubtypic influenza virus in mice immunized with cGAMP adjuvanted H7N9 vaccine, we examined the specific T cell immune response against the NP, which was a highly conserved antigen with a high proportion in the whole virus inactivated vaccine. We found that intranasal co-administration of H7N9 vaccine with cGAMP could induce high levels of NP-specific IFN-γ-producing T cells, especially large amounts of IFN-γ-producing CD4^+^ T cells, suggesting a considerable Th1 response. Previous studies by Ivana et al. using OVA antigen supplemented with cGAMP also suggested that cGAMP is an adjuvant that induces a Th1 biased response (16). In addition, recent studies have shown that cGAMP acts as a potent adjuvants *in vitro* and *in vivo*, enhancing the induction of functional antigen-specific CD8^+^ T cell responses in mice and human cells (32). By further detecting the expression of IFN-γ and granzyme B in the lung tissue of immunized mice in an early stage (after receiving a heterologous influenza virus challenge), it was found that the cGAMP adjuvant group had more cytotoxic effector molecules in mice lung tissue in short-term after virus infection. These observations demonstrate that the cross-reactive NP-specific CD8^+^ T and CD4^+^ T cells can rapidly proliferate, differentiate, and be recruited to infection sites after viral infection, kill viral infected cells through direct killing, FasL, dependent, IFN-γ dependent or TRAIL dependent pathway and mediate clearance of virus (33–36).

In the present study, we showed that intranasal delivery of an inactivated H7N9 vaccine by using cGAMP as a mucosal adjuvant induces both systemic and mucosal immunity with an antigen dose-sparing effect. In addition, the cGAMP adjuvanted vaccine elicited high HAI antibody responses and effective protection against homologous viral challenge. Furthermore, the administration of cGAMP-vaccine induced a enhanced cross-reactive T cell responses that conferred cross protection against heterosubtypic influenza A virus challenges. Our results collectively suggest that the cGAMP may be a promising adjuvant for vaccines targeted against pandemic influenza and a mucosal vaccine for broad protection against divergent influenza A virus.

## Data Availability Statement

All datasets generated for this study are included in the manuscript/Supplementary Files.

## Ethics Statement

The protocol for the animal study (Protocol Number: 17-1250) was approved by the laboratory animal management committee, and the laboratory animal ethics and welfare protection group of Shanghai Institute of Biological Products. All animal procedures were carried out in accordance with the animal ethics guidelines of the Chinese National Health and Medical Research Council (NHMRC).

## Author Contributions

JL performed the majority of the experiments, evaluated the data, prepared the manuscript. XL and FX participated in lung virus titration. FG, YY, and MZ participated in immune response assay. ZC and WT conceived and designed the experiments, revised the manuscript.

### Conflict of Interest

JL, FX, FG, YY, MZ, and ZC were employed by company Shanghai Institute of Biological Products Co., Ltd. The remaining authors declare that the research was conducted in the absence of any commercial or financial relationships that could be construed as a potential conflict of interest.

## Supplementary Material

Figure S1 SDS-PAGE analysis of inactivated whole-virion h7n9 influenza vaccine. The bulk of inactivated whole-virion H7N9 influenza vaccine with a total protein contentation of 400 ug/ml was treated with PNGase F, the optimal ratio of PNGase F to bulk was 1:50(v/v). After treatment, the vaccine sample was fractionated by SDS-PAGE under reducing conditions and stained with Coomassie Brilliant Blue.

Figure S2 Histologic analysis of mouse lung tissues after intranasal administration with cGAMP as adjuvant. Mice were intranasally administered once with with 5 μg **(A)**, 25μg **(B)**, 50 μg **(C)**, 100 μg **(D)** cGAMP or 1.5 μg inactivated whole-virion H7N9 influenza vaccine with or without 5 μg cGAMP **(E,F)**, the PBS **(G)** and LPS **(H)** groups were used as negative control and lung injury control, respectively. The lung tissues were removed for hematoxylin and eosin (HE) staining 24 h after administration. Representative photos are shown with 3 mice in each groups. Scale bar denotes 100 μm.
