# Supplementary material for: Enhancing Immune Response and Heterosubtypic Protection Ability of Inactivated H7N9 Vaccine by Using STING Agonist as a Mucosal Adjuvant
Source: Front Immunol. 2019 Sep 27;10:2274. doi: 10.3389/fimmu.2019.02274 (PMC6777483; doi:10.3389/fimmu.2019.02274)
Supplement: Supplementary file 1 [file Table_1.DOCX]

**Supplementary Materials and Methods**

**SDS-PAGE analysis of whole-virion H7N9 influenza vaccine**

The bulk of inactivated whole-virion H7N9 influenza vaccine with a total protein contentation of 400ug/ml was treated with PNGase F (New England biolabs), the optimal ratio of PNGase F to bulk was 1:50(v/v), followed by incubation at 37℃ overnight, the deglycosylated vaccine sample was fractionated by 12% SDS-PAGE under reducing conditions and stained with Coomassie Brilliant Blue R-250 according to standard protocols.

**Histological analysis of lung tissue**

The lung tissues were taken out from 3 mice in each group at 24h post immunization. For histological examinations staining, the lung tissue was fixed in 4% formaldehyde, dehydrated using a graded ethanol series, embedded in paraffin blocks and cut into 10 μm sections which were stained with hematoxylin and eosin (H&E) according to standard methods. Finally, the histopathologic changes of the lung tissues were examined and observed under licit microscope.
