# Supplementary figures and images for: Enhancing Immune Response and Heterosubtypic Protection Ability of Inactivated H7N9 Vaccine by Using STING Agonist as a Mucosal Adjuvant
Source: Front Immunol. 2019 Sep 27;10:2274. doi: 10.3389/fimmu.2019.02274 (PMC6777483; doi:10.3389/fimmu.2019.02274)

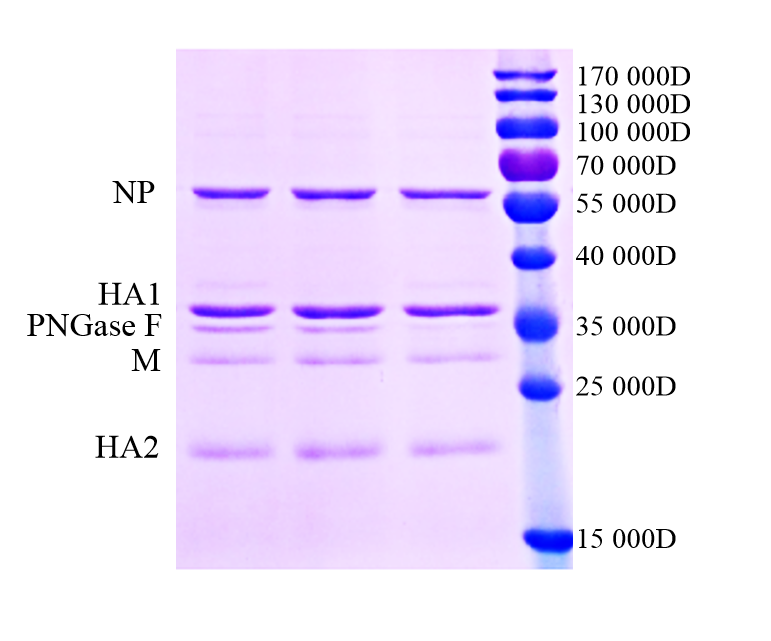

Supplement: Figure S1 — SDS-PAGE analysis of inactivated whole-virion h7n9 influenza vaccine. The bulk of inactivated whole-virion H7N9 influenza vaccine with a total protein contentation of 400 ug/ml was treated with PNGase F, the optimal ratio of PNGase F to bulk was 1:50(v/v). After treatment, the vaccine sample was fractionated by SDS-PAGE under reducing conditions and stained with Coomassie Brilliant Blue. [file Image_1.TIF]

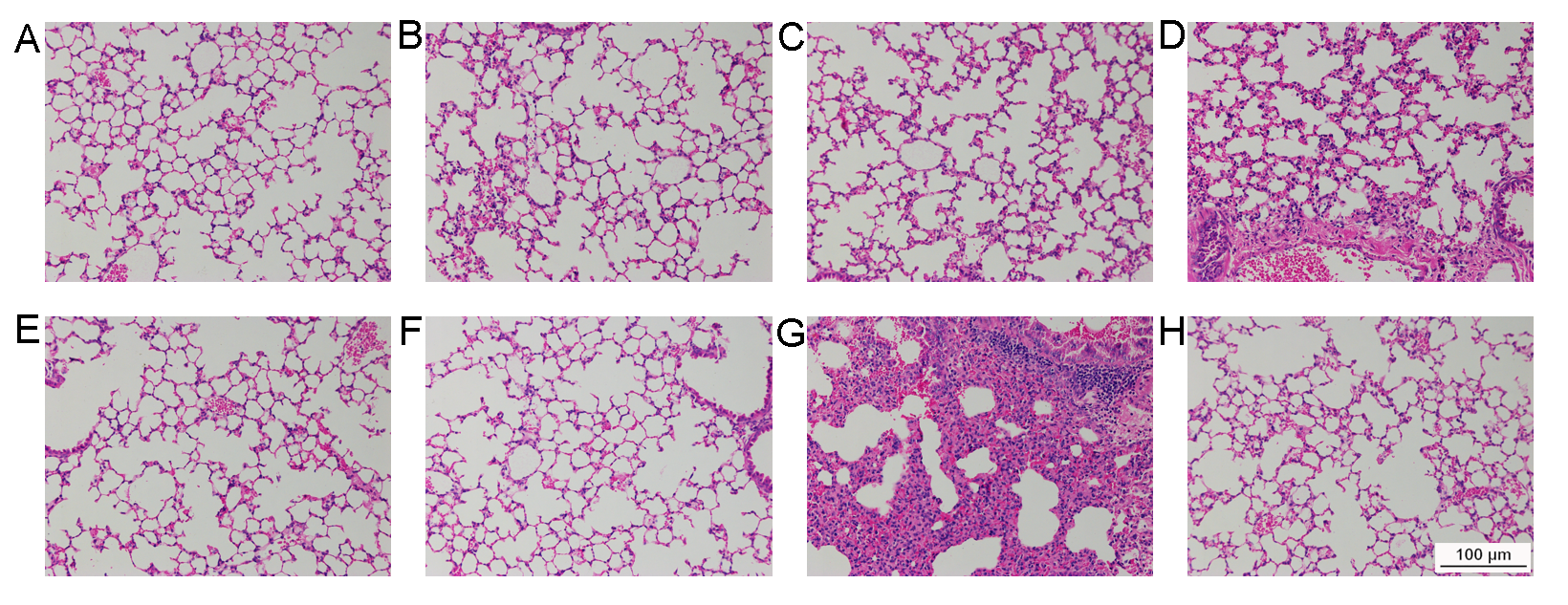

Supplement: Figure S2 — Histologic analysis of mouse lung tissues after intranasal administration with cGAMP as adjuvant. Mice were intranasally administered once with with 5 μg (A), 25μg (B), 50 μg (C), 100 μg (D) cGAMP or 1.5 μg inactivated whole-virion H7N9 influenza vaccine with or without 5 μg cGAMP (E,F), the PBS (G) and LPS (H) groups were used as negative control and lung injury control, respectively. The lung tissues were removed for hematoxylin and eosin (HE) staining 24 h after administration. Representative photos are shown with 3 mice in each groups. Scale bar denotes 100 μm. [file Image_2.TIF]
